# Genetic Variation in the *Plasmodium falciparum* Circumsporozoite Protein in India and Its Relevance to RTS,S Malaria Vaccine

**DOI:** 10.1371/journal.pone.0043430

**Published:** 2012-08-17

**Authors:** Mohammad Zeeshan, Mohammad Tauqeer Alam, Sumiti Vinayak, Hema Bora, Rupesh Kumar Tyagi, Mohd Shoeb Alam, Vandana Choudhary, Pooja Mittra, Vanshika Lumb, Praveen Kumar Bharti, Venkatachalam Udhayakumar, Neeru Singh, Vidhan Jain, Pushpendra Pal Singh, Yagya Dutta Sharma

**Affiliations:** 1 Department of Biotechnology, All India Institute of Medical Sciences, New Delhi, India; 2 Regional Medical Research Centre for Tribals, Jabalpur, Madhya Pradesh, India; 3 Malaria Branch, Division of Parasitic Diseases and Malaria, Center for Global Health, Centers for Disease Control and Prevention, Atlanta, Georgia, United States of America; Museum National d'Histoire Naturelle, France

## Abstract

RTS,S is the most advanced malaria vaccine candidate, currently under phase-III clinical trials in Africa. This *Plasmodium falciparum* vaccine contains part of the central repeat region and the complete C-terminal T cell epitope region (Th2R and Th3R) of the circumsporozoite protein (CSP). Since naturally occurring polymorphisms at the vaccine candidate loci are critical determinants of the protective efficacy of the vaccines, it is imperative to investigate these polymorphisms in field isolates. In this study we have investigated the genetic diversity at the central repeat, C-terminal T cell epitope (Th2R and Th3R) and N-terminal T cell epitope regions of the CSP, in *P. falciparum* isolates from Madhya Pradesh state of India. These isolates were collected through a 5-year prospective study aimed to develop a well-characterized field-site for the future evaluation of malaria vaccine in India. Our results revealed that the central repeat (63 haplotypes, n = 161) and C-terminal Th2R/Th3R epitope (24 haplotypes, n = 179) regions were highly polymorphic, whereas N-terminal non-repeat region was less polymorphic (5 haplotypes, n = 161) in this population. We did not find any evidence of the role of positive natural selection in maintaining the genetic diversity at the Th2R/Th3R regions of CSP. Comparative analysis of the Th2R/Th3R sequences from this study to the global isolates (n = 1160) retrieved from the GenBank database revealed two important points. First, the majority of the sequences (∼61%, n = 179) from this study were identical to the Dd2/Indochina type, which is also the predominant Th2R/Th3R haplotype in Asia (∼59%, n = 974). Second, the Th2R/Th3R sequences in Asia, South America and Africa are geographically distinct with little allele sharing between continents. In conclusion, this study provides an insight on the existing polymorphisms in the CSP in a parasite population from India that could potentially influence the efficacy of RTS,S vaccine in this region.

## Introduction

Malaria, especially that caused by *Plasmodium falciparum* is responsible for nearly 800,000 deaths each year worldwide, most of whom are young children in Sub-Saharan Africa [1]. Approximately 1.5 million cases of malaria are reported in India each year, of which 50% are due to *P. falciparum* [1]. Given the high impact of malaria on human health, a highly effective vaccine is definitely needed for long-term control, elimination and possible eradication of malaria.

As of today there is no licensed vaccine against malaria. However, a number of potential vaccine candidates targeted against pre-erythrocytic, erythrocytic and sexual stages of *P. falciparum* are under various stages of clinical development [2]. The most advanced among all, is RTS,S, a pre-erythrocytic stage vaccine based on the parasite's circumsporozoite protein (CSP) [3]. The CSP is the most abundant protein on sporozoite surface and consists of a highly polymorphic central repeat region flanked by a less polymorphic N-terminal and highly polymorphic C-terminal non-repeat regions [4]. The central region, which is predominantly consisting of tandem repeats of NANP (N, Asparagine; A, Alanine and P, Proline), in addition to small number of NVDP (N, Asparagine; V, Valine; D, Aspartic acid and P, Proline) repeats, constitutes immunodominant B cell epitopes. Whereas the C-terminal region, which is concentrated in two sub-regions, called Th2R and Th3R, makes both B cell and T cell epitopes. In RTS,S recombinant vaccine, 19 NANP repeats and entire C-terminal sequence of the CSP from NF54/3D7 *P. falciparum* strain (amino acid residue 207 to 395) are fused to the hepatitis B surface antigen (HBsAg), which in turn is co-expressed with additional unfused HBsAg in *Saccharomyces cerevisiae* yeast [5].

Since 1992, when the first trial of RTS,S, was conducted, it has progressed through multiple phase-I and II trials on children and infants in several African countries [3], [6]–[9]. After obtaining substantial level of protective efficacies in phase II trials, which ranged from 30 to 50%, it is currently going through large-scale phase-III trials at 11 sites in seven countries in Africa [3], [10]–[15]. In fact, initial results of the phase-III trials have been published recently showing that the RTS,S vaccine provide African children aged 5 to 17 months with significant protection against clinical (56%) and severe (47%) malaria [16]. This study marks an important milestone in the development of malaria vaccine, and there is a hope that the first generation malaria vaccine will be licensed by the year 2015. The malaria vaccine community has set a goal to license a safe and affordable vaccine by 2025, that has >80% efficacy and lasts longer than four years [17].

The occurrence of high genetic diversity in the malaria parasite, especially at the surface-expressed molecules poses the greatest challenge in developing a universally effective malaria vaccine. Almost all *P. falciparum* antigens currently under consideration for vaccine development including CSP have been observed to exhibit polymorphisms in field isolates from various malaria-endemic regions of the world [18]–[25]. In addition, polymorphisms in the CSP has been shown to restrict T cell reactivity to specific epitope and affect binding to HLA indicating selection of variants due to immune pressure [26], [27]. Given the importance of antigenic diversity in influencing the outcome of any vaccine, it is very important to characterize the prevailing level of variation in CSP in different endemic regions as this will help to determine if vaccine escape variants will compromise the efficacy of RTS,S vaccine. Therefore, we have conducted a five-year prospective cohort study in the Madhya Pradesh state of India, to examine the genetic polymorphisms both at the central repeat and C-terminal regions of the CSP, included in RTS,S, and also at the N-terminal T cell epitope region. The data from this study were compared with the sequences available for the *P. falciparum* isolates from other malaria endemic countries in the world. The global distribution of various allelic forms of the CSP has also been discussed.

## Materials and Methods

### Ethics Statement

The study was conducted in accordance with the procedure and guidelines approved by the Indian Council of Medical Research (ICMR), Government of India, and with ethical approval from the institutional review board of the All India Institute of Medical Sciences (AIIMS), New Delhi, Regional Medical Research Centre for Tribals (RMRCT), Jabalpur, India, and the Centers for Disease Control and Prevention, Atlanta, U.S.A. Written informed consent was obtained from each participant or their parents or guardians before being included in this study.

### Study design, study sites and sample collection

**Figure 1:**
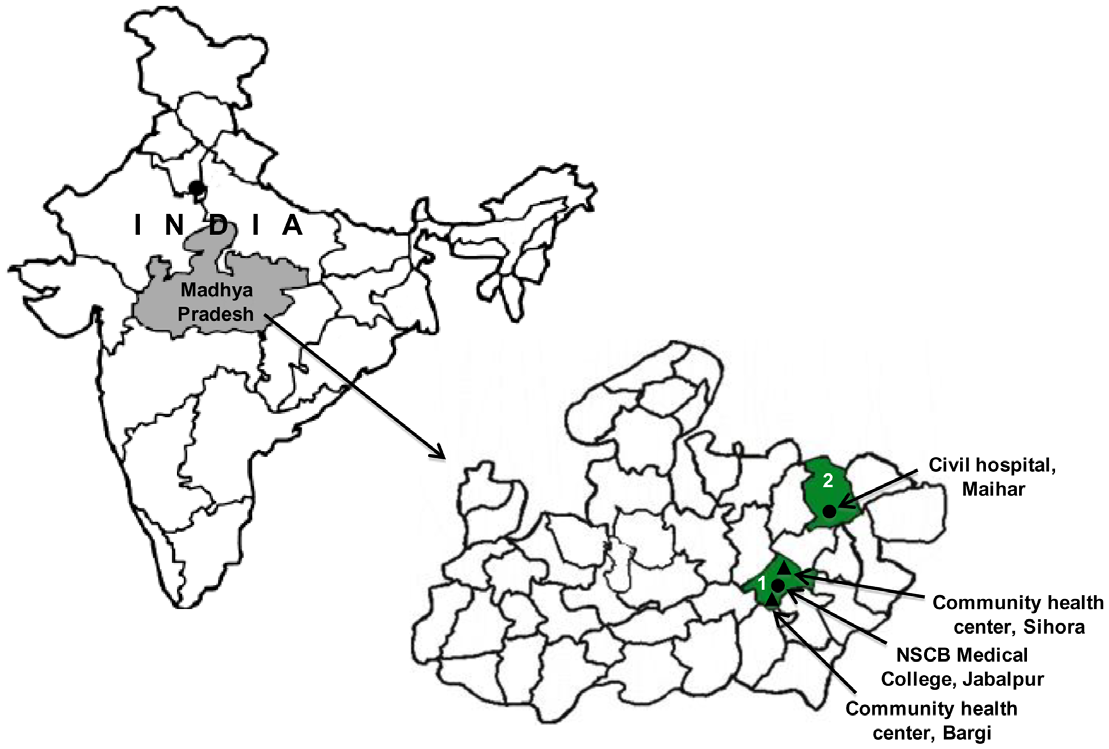
Map of India showing sample collection sites. Map showing the study sites in Jabalpur (marked as 1) and Satna (marked as 2) districts of Madhya Pradesh, India. Solid triangles indicate the community cohort sites whereas hospital cohort sites are shown as solid circles.

This five-year prospective study (2005–2010) was designed to develop a well-characterized field site, where the epidemiology of malaria, immune responses to key parasite antigens, genetic diversity at leading vaccine candidate antigens, and *Anopheles* vector characteristics could be understood. There were two cohort groups in this study as the source of our samples namely: the hospital-based cohort and the community-based cohort. The hospital-based cohort group represents the patients who attended either the Netaji Subhash Chandra Bose (NSCB) Medical College at Jabalpur or the Civil Hospital Maihar in Satna district. The community-based cohort group represents patients who were enrolled from study sites in Bargi and Sihora; both in the Jabalpur district (**Fig** **1**). Approximately, 200–300 µl of peripheral blood samples were collected from each patient who had fever or fever-like symptoms at the time of enrollment. The malaria epidemiology in both the Jabalpur and Satna districts, which are 150 kilometers apart, are identical with transmission level varying from hypo-endemic to meso-endemic. The details of patient enrollment, sample collection and other epidemiological parameters are available (**Text S1**).

### Genomic DNA extraction and amplification of csp gene

Genomic DNA was extracted from *P. falciparum* infected blood using Genomic DNA Extraction Kit (Bioneer Corporation, Korea), in accordance with the manufacturer's protocol. The CSP1forward (5′-TTAGCTATTTTATCTGTTTCTTCC-3′) and CSP2 reverse (5′- TAAGGAACAAGAAGGATAATACC-3′) primers designed using 3D7 strain as a reference sequence, were used to amplify 1177 bp of the 1194 bp complete pfcsp gene. The PCR cycling conditions for this primer pair were: 10 minutes initial denaturation at 94°C followed by 35 cycles with 1 minute denaturation at 94°C, 1 minute annealing at 57°C, 90 seconds extension at 72°C and a final 10 minute extension at 72°C. The resulting PCR products were diluted 1∶10 and 2 μl of this was used as a template to amplify the internal 1026 bp fragment using CSP3 forward (5′-GAAATGAATTATTATGGGAAACAG-3′) and CSP4 reverse (5′-GAAGGATAATACCATTATTAATCC-3′) primers. The 1026 bp fragment encompassed the N-terminal T cell epitope, central repeat and C-terminal T ell epitope regions. The PCR cycling conditions for CSP3/CSP4 primer pair were: 10 minutes initial denaturation at 94°C followed by 35 cycles with 1 minute denaturation at 94°C, 40 seconds annealing at 57°C, 80 seconds extension at 72°C and a final 10 minute extension at 72°C. Proof reading polymerase *Pfx* (Invitrogen Life Sciences, Carlsbad, CA, USA) was used to avoid introduction of any error during PCR. Care was also taken to exclude the possibility of cross-contamination where a negative control without the template DNA was always used. Further, the DNA from a culture adapted *P.falciparum* was used as a control to check the reliability of the sequencing. The amplified products were resolved on 1.2% agarose gel.

### Sequencing of the amplified products and sequence analysis

The desired sized bands were excised from the gel and purified using the Gel Extraction Kit (Bioneer Corporation), in accordance with the manufacturer's protocol. The methods for cycle sequencing PCR and cleanup were same as described earlier [28]. The products were sequenced on both strands using CSP3 forward, CSP4 reverse and CSP-D reverse (5′- TGGGTCATTTGGCATATTGTG-3′) primers, using ABI Big Dye Terminator Ready Reaction Kit Version 3.1 (PE Applied Biosystems, CA, USA) on an ABI-310 genetic analyzer (ABI 310 Genetic Analyzer; PE Applied Biosystems, CA, USA). The BioEdit Sequence Alignment Editor [29] and GeneDoc-Version 2.6.002 [30] were used to analyze the sequencing electropherograms and generate sequence alignment, respectively. The *csp* sequences of the eight laboratory-adapted *P. falciparum* strains (Dd2, K1, MAD20, Wellcome, 7G8, HB3, 3D7, and RO33) were also included in the alignment to make comparison. All of these sequences generated in this study have been submitted into the NCBI GenBank database under the accession numbers HM756094-HM756109 and HM582036-HM582081.

We also compared our sequences with all the published and unpublished CSP sequences deposited in the NCBI GenBank database from around the world. The details about these isolates have been provided as (**Table S1**). Here we could only compare C-terminal Th2R/Th3R sequences since this region of the CSP has most widely been sequenced. The genetic relationship among the global CSP sequence (Th2/Th3R) haplotypes was deduced by the algorithm Minimal Spanning Tree (MST), implemented in BioNumerics version 6.6 (Applied-Maths, Inc. Austin, TX). In this algorithm, the haplotype with the highest numbers of single locus variants (SLVs) is considered as a root haplotype and all other haplotypes as relatives. This not by any means suggests the origin or ancestry of a particular haplotype.

### Statistical analysis

The different parameters of genetic diversity such as numbers of haplotypes (H), segregating sites (S), haplotype diversity (Hd) and average number of nucleotide differences per site between two sequences (π), for the isolates from each country were calculated using DnaSP ver. 4. 10. 9 [31]. Only Th2R/Th3R region sequences were included in these analyses. The difference between non-synonymous (dN) and synonymous (dS) mutations were estimated in MEGA version 4.0 [32] using the method of Nei and Gojobori's [33] with the Jukes and Cantor (JC) correction to test the evidence of positive (balancing) selection. We also performed Fu & Li's F* [34] and Tajima's D [35] test statistics to test the neutral theory of evolution using DnaSP. In order to look for pattern of selection across the Th2R/Th3R region, a sliding window analysis of π, Fu & Li's F* and Tajima's D were performed with window length of 10 bp and step size of 5 bp. The significant positive values for dN-dS, Fu & Li's F*and Tajima's D indicate positive (balancing) selection whereas negative values indicate negative (purifying) selection. The Fu & Li's F* and Tajima's D values are also affected by demographic factors including population size expansion or contraction, whereas the dN-dS is insensitive to these factors.

## Results

A total of 2336 (n = 603, Hospital cohort; n = 1733, Community cohort) patients enrolled over the 5 years period based on the adopted inclusion criteria. By light microscopy, only 780 (n = 603 Hospital cohort; n = 177 Community cohort) patients were confirmed to have *P. falciparum* infection. Of them, 626 samples were subjected to PCR amplification of *pfcsp* gene while remaining 154 samples were not available (Details of these patients are given in **Table** **S2**). A total of 216 samples gave PCR amplification for this gene. The PCR positivity rate for *pfcsp* gene was lower (34.5%) as compared to other vaccine candidate antigen genes (44% to 56%) and *P.falciparum* dihydrofolate reductase (*pfdhfr*) gene (66%) among these 626 isolates (Unpublished data). Such variation in PCR positivity among the isolates can occur due to various reasons such as variable copy number of the gene, annealing efficiency of the primers, level of parasitemia in the samples etc. Successful sequencing data was obtained from 161 samples each for the N-terminal T cell epitope and central repeat regions (n = 126 Hospital cohort; n = 35 Community cohort), while 179 samples provided sequence data for the C-terminal T cell epitope region (n = 142 Hospital cohort; n = 37 Community cohort) (**Table** **1**). All the samples were of mono-infections as checked by the PCR analysis of the merozoite surface protein (*msp1* and *msp2*) genes (Data not shown). Further, there were no mixed peaks in *pfcsp* gene sequences for any of the isolate which also ruled out the possibility of mixed infections with other *P.falciparum* strains.

**Table 1:** Year-wise distribution of *P. falciparum* isolates collected from two sites for CSP sequence analysis.

Cohort Groups	Number of samples sequenced for csp gene																	
	Central repeat region						N-terminal region						C-terminal region					
	2005	2006	2007	2008	2009	Total	2005	2006	2007	2008	2009	Total	2005	2006	2007	2008	2009	Total
**Hospital**	4	33	64	4	21	126	4	33	64	4	21	126	4	30	80	5	23	142
**Community**	0	0	21	2	12	35	0	0	21	2	12	35	0	1	21	1	14	37
**Total**	4	33	85	6	33	161	4	33	85	6	33	161	4	31	101	6	37	179

### Sequence diversity in the central repeat region

**Figure 2:**
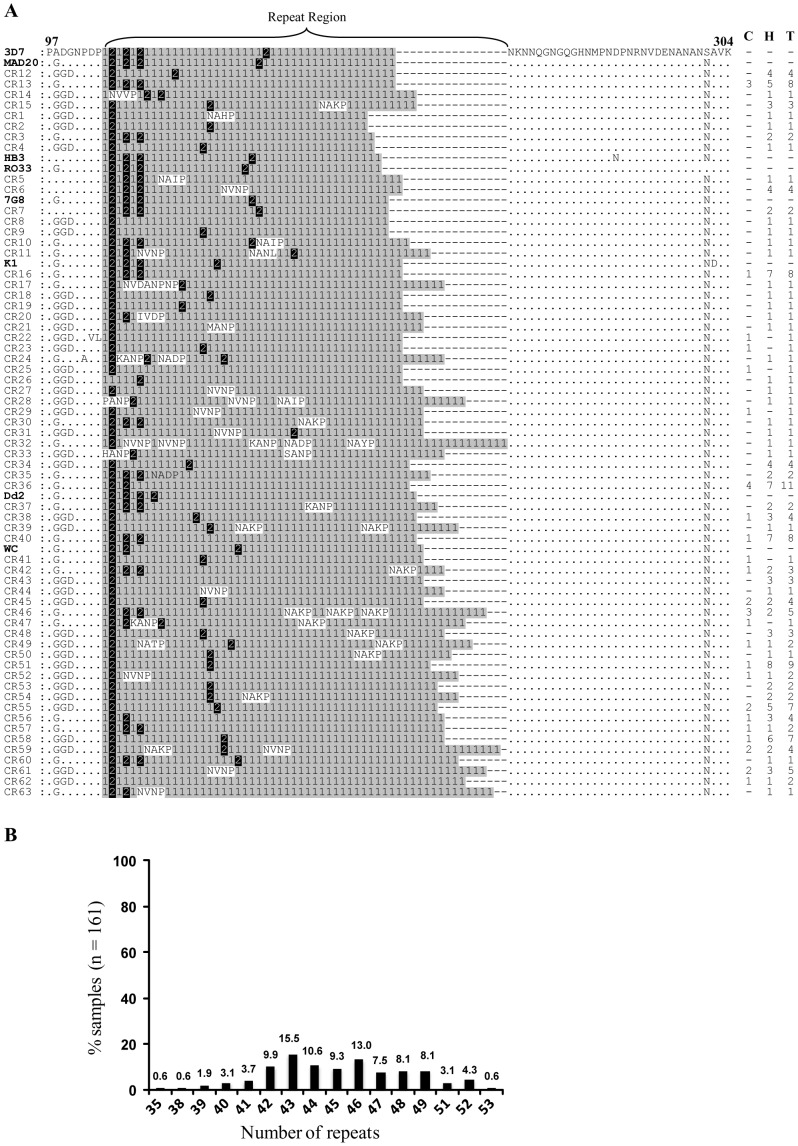
Sequence diversity in the central repeat region of PfCSP. (**A**) Representation of the variation in sequence repeats in the central region of the CSP in 161 samples. The sequences of eight laboratory-adapted *P. falciparum* strains [Dd2 (Indochina), K1 (Thailand), MAD20 (Papua New Guinea), Wellcome (West Africa), 7G8 (Brazil), HB3 (Honduras), 3D7 (The Netherlands) and RO33 (Ghana)] are shown here for comparison. The NANP repeats are indicated as “1 with gray shade", NVDP repeats are indicated as “2 with black shade" and all other repeats are un-shaded. Numbers on the right indicate numbers of samples belonging to that particular haplotype. Numbers above the alignment are amino acid position with reference to 3D7 sequence. Dots represent amino acid positions identical to the 3D7 haplotype, whereas those different are indicated. Dashes have been inserted for maximum alignment. C, community cohort; H, hospital cohort; T, total; WC, Wellcome. (**B**) Distribution of repeats in the central region of the CSP in 161 samples.

Sixty-three unique haplotypes (CR1 to CR63) were observed among 161 isolates in the central repeat region (**Fig** **2A**). The number of tetrapeptide repeats that includes NANP and other minor variants, in this region varied from 35 to 53 among these haplotypes. Approximately 58% of the samples (n = 161) had number of tetrapeptide repeats between 42 and 46 (**Fig** **2B**). Six haplotypes were exclusive to community cohort, 18 were common to both community and hospital cohorts, while remaining 39 were only found in hospital cohort. Sixteen samples (CR12 to CR15) had same number of repeats (number of repeats  = 42) as in 3D7 strain, however, they had variation in sequences (**Fig** **2A**). None of the samples were identical to either 3D7 or Dd2 strain sequences at this locus.

### Sequence diversity in the N-terminal non-repeat region

**Figure 3:**
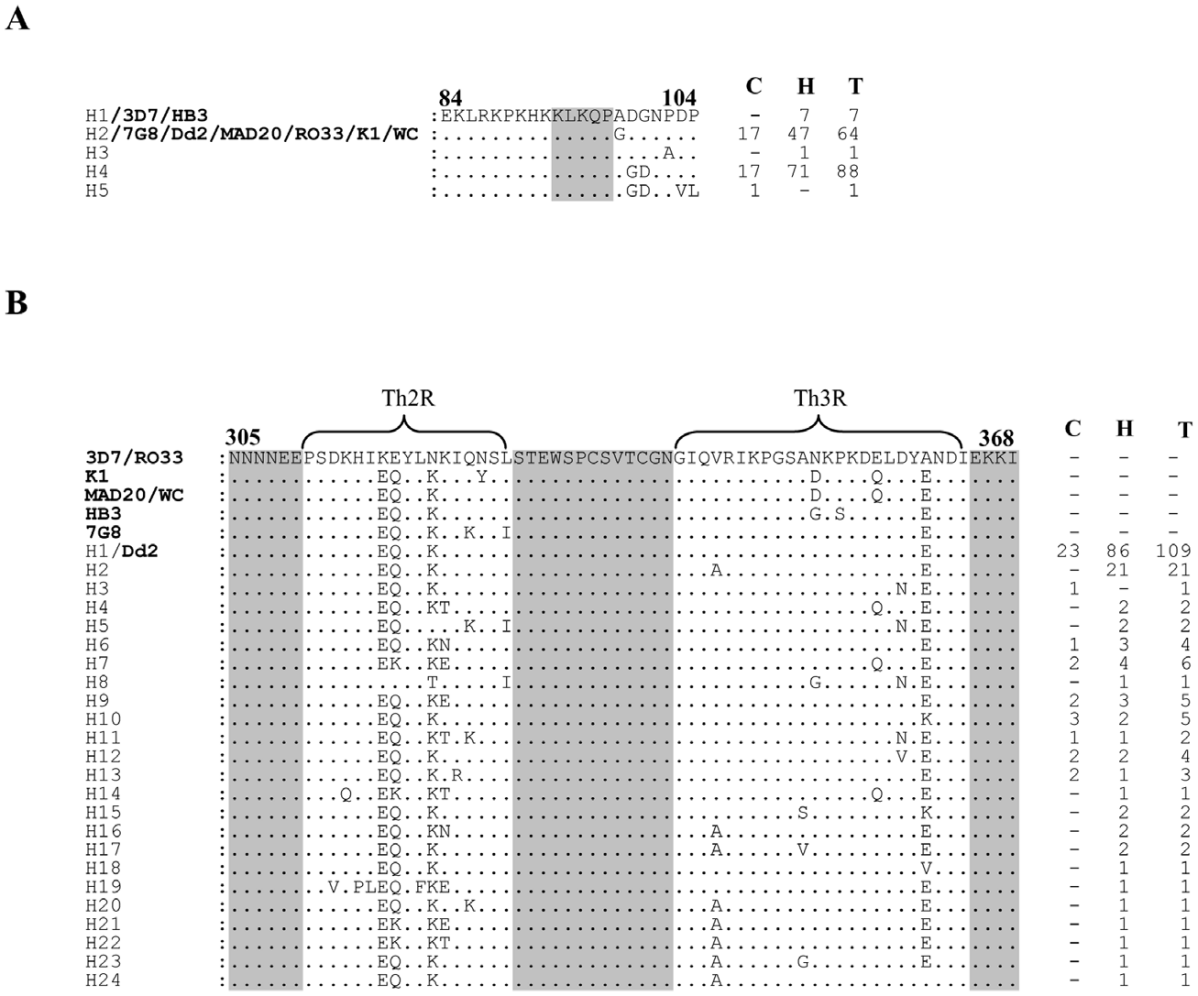
Sequence diversity in the N- and C-terminal non-repeat region of PfCSP. (**A**) Sequence alignment showing polymorphisms in the non-repeat N-terminal T cell epitope region (amino acid residue 84 to 104) of CSP in 161 samples. The shaded region (amino acid residue 93 to 97) is a conserved motif involved in sporozoite invasion of mosquito salivary gland as well as in binding to hepatocytes prior to invasion. (**B**) Sequence alignment showing polymorphisms in the non-repeat C-terminal T cell epitope regions (Th2R spanning from amino acid residues 311 to 327 and Th3R from amino acid residues 341 to 364) of CSP in 179 samples. The highly conserved sequences flanking the Th2R and Th3R domains are shaded grey. The eight laboratory-adapted strains are also included in this alignment. Numbers on the right indicate numbers of samples belonging to that particular haplotype. Dots represent amino acid positions identical to the 3D7 haplotype, whereas those different are indicated. C, community cohort; H, hospital cohort; T, total; WC, Wellcome.

As expected the N-terminal non-repeat region was highly conserved among the samples analyzed and resulted into only five haplotypes-H1 to H5 (**Fig** **3A**). The H4 and H2 haplotypes observed in ∼55% (n = 161) and ∼40% (n = 161) samples respectively, were predominantly present in our study sites. The H2 haplotype was identical to 7G8, Dd2, MAD20, RO33, K1 and Wellcome strain sequences at this locus. The H1 haplotype observed in seven samples was identical to 3D7 and HB3 sequences. The haplotype H4 was exclusively found in this study.

### Sequence diversity in the C-terminal non-repeat region

A total of 24 Th2R/Th3R sequence haplotypes (H1 to H24; 1 specific to community cohort, 15 specific to hospital cohort and 8 common to both cohorts) were defined from 179 samples analyzed, predominant being the H1/Dd2 type sequence (∼61%, n = 179) (**Fig** **3B**). This most common haplotype H1 differed from 3D7 sequence at 4 codons (3 in the Th2R and 1 in the Th3R region). The second most common haplotype H2 (∼12%, n = 179) found only in hospital cohort samples was a single locus variant (SLV) of H1, since it differed from H1 only by one mutation (Valine to Alanine) in the Th3R region. In comparison to 3D7 sequence that is represented in RTS,S vaccine, polymorphism was observed at 13 codons in the Th2R region and at 7 codons in the Th3R region (**Fig** **3B**). None of the sample had Th2R/Th3R sequence identical to the 3D7 strain. The over all haplotype diversity (Hd) for the combined Th2R/Th3R region was 0.614±0.041 and π nucleotide diversity was 0.0065±0.0065, suggesting moderate level of genetic diversity at the C-terminal region of CSP in this population (**Table** **2**). The evidence of selection occurring on this gene was not very conclusive as both the Fu & Li's F* (−3.53, *P*<0.05) and Tajima's D (−2.13, *P*<0.05) were negative for the whole Th2R/Th3R region. Sliding window analysis also showed negative values of these indices across the shorter segments of the Th2R and Th3R (**Fig** **S2**). However, the dN-dS difference (0.008±0.003) was positive for this region.

**Table 2:** Nucleotide diversity and tests of neutrality for the C-terminal epitope region (Th2R/Th3R) of the *P. falciparum* csp gene in global isolates.

Country	N	H	S	Hd±SD	π±SD	dN-dS±SE	Fu & Li's F*	Tajima's D	Reference
**Asia**									
Jabalpur (India)	179	24	26	0.614±0.041	0.0065±0.0065	0.008±0.003	−3.53***	−2.13***	This study
India	12	4	6	0.561±0.154	0.0073±0.0025	0.004±0.006	−1.55*	−1.53*	[22]
Iran	90	5	3	0.603±0.041	0.0056±0.0004	0.007±0.004	1.23*	1.54*	[42]
Myanmar	21	5	9	0.486±0.124	0.0081±0.0028	0.010±0.004	−1.01*	−1.24*	[40], [52]
Thailand	336	12	16	0.629±0.018	0.0116±0.0006	0.015±0.005	−0.77*	−0.37*	[38]–[40]
Vietnam	142	20	14	0.697±0.040	0.0105±0.0012	0.013±0.004	0.43*	−0.79*	[55]
Indonesia	36	5	8	0.260±0.095	0.0033±0.0013	0.004±0.002	−1.78*	−1.92***	[55]
Vanuatu	136	2	2	0.312±0.042	0.0032±0.0004	0.004±0.002	0.91*	1.03*	[21]
PNG	22	1	0	0.000±0.000	0.0000±0.0000	0.000±0.000	0	0	[56]
Total	974	53	33	0.636±0.017	0.0094±0.0004	0.012±0.004	−2.64***	−1.62*	
**South America**									
Venezuela	10	7	14	0.911±0.077	0.0333±0.0034	0.036±0.015	0.85*	0.64*	[22]
Brazil	33	3	5	0.316±0.093	0.0077±0.0022	0.009±0.004	1.12*	0.56*	[52], [53]
Peru	138	3	7	0.456±0.032	0.0116±0.0008	0.015±0.007	1.61*	1.71**	[54]
Total	181	10	16	0.506±0.036	0.0138±0.0011	0.017±0.007	−0.12*	−0.37*	
**Africa**									
Kenya	18	13	18	0.928±0.052	0.0302±0.0039	0.034±0.011	−0.16*	−0.16**	[22]
The Gambia	47	23	19	0.957±0.014	0.0318±0.0020	0.040±0.010	1.08*	0.59**	[41], [57]
Senegal	11	9	10	0.964±0.051	0.0235±0.0029	0.030±0.001	1.10*	0.47**	[52]
Sierra Leone	99	40	27	0.919±0.017	0.0253±0.0012	0.027±0.009	−0.49*	−0.47*	[58]
Cameroon	9	7	12	0.944±0.070	0.0236±0.0053	0.030±0.001	−0.81*	−0.84*	[22]
Total	184	63	30	0.959±0.006	0.0280±0.0009	0.033±0.010	−1.09*	−0.30*	

*Note:* N, Number of isolates analyzed from each country; H, Number of haplotypes; S, Number of segregating (polymorphic) sites; Hd, Haplotype diversity; π, Observed average pairwise nucleotide diversity; dN-dS, rate of non-synonymous mutations minus rate of synonymous mutations; PNG, Papua New Guinea; SD, Standard deviation; SE, Standard error. *, *P*>0.10; **, *P*>0.05; ***, *P*<0.05.

### Global analysis of the Th2R/Th3R sequences

**Figure 4:**
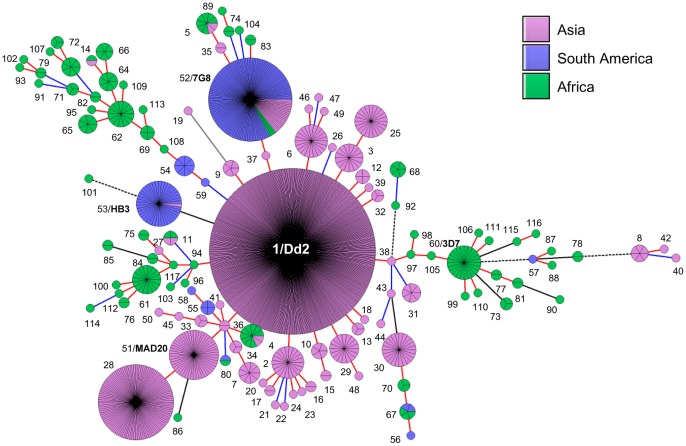
Global population structure of csp gene. A minimal spanning tree (MST) generated using BioNumerics software version 6.6 showing the relationship among all the 117 haplotypes based on the Th2R/Th3R sequences of the CSP from worldwide isolates [Asia, n = 974; South America, n = 181 and Africa, n = 184)]. Each circle represents an individual haplotype and the size of the circle is proportional to the number of isolates belonging to that haplotype (also shown as pie). The lines connecting the circles are branch length and are red if two haplotypes differ by only one mutation, blue if differ by 2 mutations, solid black if differ by 3 mutations, dashed black if differ by 4 mutations and gray if they differ by more than 4 mutations. Numbers outside the circles indicate haplotypes H1 to H117. The Dd2, 3D7, HB3, 7G8 and MAD20 type sequences are highlighted in bold. The haplotype pairs H55 & H58; H57 & H87; H60 & H106 and H97 & H98 are identical at amino acid level; but have one synonymous mutation. H1 to H24 are the same haplotypes we observed in our study sites and shown in **Fig** **3B**. Please refer to **Table S1, Table S3** and **Fig S1** for more details on these sequence haplotypes and their country-wise distributions.

The multiple sequence alignment and minimal spanning tree (MST) analysis of Th2R/Th3R sequences of all 1339 global isolates including 179 from the current study resulted into 117 unique haplotypes (**Table** **2**, **Fig** **4**, **Fig S1**). These included 53 haplotypes from Asia (n = 974), 10 haplotypes from South America (n = 181) and 63 haplotypes from Africa (n = 184). Other parameters of genetic diversity also suggested that the African populations show greater diversity at the Th2R/Th3R locus followed by Asian and South American populations (**Table** **2**). The level of genetic diversity at the Th2R/Th3R locus in our study population was very close to the diversity shown by the isolates from other Asian countries. Broadly, there were two predominant Th2R/Th3R sequence haplotypes in South America, one was H52 (7G8 type) and other was H53 (HB3 type) (**Fig** **4**). In Asia, five Th2R/Th3R sequence haplotype groups can be described. The first group includes H1 (Dd2 type) and its SLVs (directly connected to H1 by red line in **Fig** **4**). The second group includes H51 (MAD20 type) sequence and its SLV H28. The third group H25 differed from the Dd2 type sequence by 2 mutations (Double locus variants or DLVs). Interestingly, the fourth group H52 was identical to the South American haplotype 7G8. The fifth group H30 was very distant from its other Asian relatives. Though there were 63 unique haplotypes in Africa, they were distributed into 3 major clusters: H60 (3D7 type), H61 and H62. The haplotype H52 (7G8 type) was the only Th2R/Th3R sequence common among Asian, African and South American population (**Fig** **4**).

## Discussion

To date, several antigens in the *P. falciparum* have been identified and considered for the development of vaccine(s) against malaria. One of the underlying challenges, which have been delaying the successful development of malaria vaccine, is the high level of antigenic diversity present in almost all candidate vaccine antigens. The antigenic diversity in the parasite arises over time as a result of immune selection pressure within the human host. Also, almost all vaccine candidates under clinical development are based on a single allelic form of the antigen and thus may not be able to provide protective immunity against all the parasite strains circulating in the population. The RTS,S, which is a subunit vaccine has shown only partial protection (about 50%) in recent phase III trials against clinical episodes of malaria [16]. It is unclear whether polymorphisms observed in the CSP is a contributing factor in the partial efficacy of this vaccine and this may become clear when follow up studies related to this vaccine trial are completed. The results from this study are relevant for future RTS,S vaccine trials in India as this is the first major study to comprehensively analyze the diversity of CSP in Indian *P.falciparum* population.

The N-terminal region of CSP, which contains T cell proliferation determinants, putative hepatocyte binding site and B cell epitopes [36], [37], did not show much polymorphisms among the 161 samples sequenced (**Fig** **3A**). This is similar to earlier studies where only limited numbers of polymorphisms have been observed within this region [21], [22], [38]–[42]. Global analysis of this region revealed that H2 (Dd2 type) is the most common haplotype found in Asia followed by H1 (3D7 type) [21], [22], [38]–[40]. Both H1 and H2 haplotypes have been previously reported from Africa and H1 being the predominant one [22], [41]. A previous study using various deletion constructs of the CSP has demonstrated that the N-terminal region of this protein contains the motif required for binding and subsequent invasion of liver cells by the sporozoites [43]. Specifically, the 82–100 amino acid residues (DNEKLRKPKHKKLKQPADG) called ‘region I-plus’ is critical and has the highest binding affinity to heparan sulfate (HS) ligand expressed on the liver cell surface [44]. Furthermore, antibodies raised against the N-terminal region have been found to be protective in nature, and were able to inhibit the binding and invasion of the liver cells by the sporozoites 45–47. Since this region is important for sporozoite attachment and invasion, the presence of non-synonymous mutations in this region may cause structural changes in the protein leading to a reduction in its binding affinity to the liver cell [45].

We observed a very high level of repeat polymorphisms in the central repeat region where almost 88% of the samples contained 42 to 49 tetrapeptide repeats (**Fig** **2A, 2B**). This is very similar to a previous study by Escalante et al where they had observed 37 to 49 repeats in 75 samples analyzed from different countries including 11 samples from India [22]. The central repeat region, apart from making immunodominant B cell epitopes also provides structural stability to CSP [22]. The simulation study by Escalante et al [22] has shown that the stability of the type-I β turn in CSP increases with the number of repeats. It is not clearly understood how the variable number of NVDP, NADP, NAHP, NVNP and other minor repeats influence antibody response against CSP. However, it has been suggested that the diversity in the repeat region is maintained by balancing selection [**22**].

Like other malaria-endemic countries we also found here high level of polymorphisms in the C-terminal region of CSP among Indian isolates. The Th2R region was more polymorphic than Th3R (**Fig** **3B**). The sequence polymorphisms in the Th3R domain are very critical as they are involved in cytotoxic T cell activity and HLA binding [26], [27]. These polymorphisms may help parasites to escape the immune pressure of the host and will have a significant impact on vaccine efficacy. However, several studies in Africa have shown that the current RTS,S vaccine induces a cross-reactive immune response against a wide range of CSP alleles and the protection is not strain-specific [48]–[50]. These studies analyzed CSP sequences of the parasites strains in the RTS,S-vaccinated individuals who became re-infected, and in the control population, and found that both groups had almost similar distribution of vaccine-type and other CSP allelic variants. This suggests that the RTS,S vaccine does not favor selection or expansion of the parasite with a particular CSP allele(s) in the vaccinated individuals. Given that the emergence and subsequent expansion of an advantageous allele in a population depends on several factors, including the strength and duration of the applied selection pressure as well as transmission dynamics in the region, it will be interesting to monitor the effect of the long-term and wide-spread use of RTS,S vaccine on the emergence of any selective CSP variants.

Interestingly, majority of our samples were identical or nearly identical to Dd2/Indochina type, and almost all samples clustered with the Asian type sequences (**Fig** **4**). As illustrated in figure 4, Th2R/Th3R sequence haplotypes are geographically distinct and have a very distinct pattern of polymorphism in populations in Asia, South America and Africa. The Dd2 type or closely related Th2R/Th3R sequences are predominant in Asia whereas 7G8 and HB3 types are predominant in South America [22], [38], [51]–[54]. As expected, Africa has lot more unique Th2R/Th3R sequence haplotypes apart from the predominant 3D7 type [22], [41]. It is also worth noting that there is not much sharing of alleles between continents at least at Th2R/Th3R region (**Fig** **4**, **Fig** **S1**). Consistent with previous study, we also found that the African populations (Hd ± SD  = 0.959±0.006) exhibit greater diversity at CSP compared to the populations from Asia (Hd ± SD  = 0.636±0.017) and South America (Hd ± SD  = 0.506±0.0036) (**Table 2**) [22]. Haplotype diversity (Hd) and average nucleotide diversity (π) in our study population was similar to that of the population from other Asian countries. We could not find any conclusive evidence of the role of positive natural selection in maintaining the diversity at CSP in Indian population. This is in agreement with previous studies where signatures of selection at CSP were not found [41]. Our analysis on the global isolates also confirms that the signature of selection at CSP is not uniform in all populations (**Table** **2** **, Fig S2**).

In conclusion, this study makes an important contribution in understanding the type and distribution of naturally occurring polymorphisms in RTS,S vaccine candidate antigen in a population from Madhya Pradesh, India, which is endemic to malaria. The N-terminal region of the CSP showed limited polymorphisms, whereas the central repeat and C-terminal regions were highly polymorphic. Almost all Th2R/Th3R sequences were identical or nearly identical to the Dd2 type or other Asian type sequences but distinct from African and South American sequences. This data would be helpful in the future trials of the RTS,S vaccine in India and to monitor changes in parasite population with different CSP variants before and after vaccine administration. Also, the global analyses of CSP allelic variants reported in this study may be helpful in identifying the predominant allele(s) prevalent in Asia, Africa and South America, and may aid in designing a region or population-specific CSP-based malaria vaccine in the future.

## Supporting Information

Figure S1 **Comparison of Th2R and Th3R sequence polymorphisms in the 1339 global isolates including 179 isolates from current study [Asia, n = 974; South America, n = 181 and Africa, n = 184)].**
(DOC)

Figure S2 **Sliding window (window size = 10** **bp, step length = 5** **bp) analysis of genetic indices π, Fu & Li**'s F* and Tajima's D across the Th2R/Th3R region of CSP in populations from (A) Asia, (B) South America and (C) Africa.
(DOC)

Text S1 **Information on participants' enrolment, characteristics, and sample collection.**
(DOC)

Table S1 **The available accession numbers, country origin and references of the isolates analyzed in this study.**
(XLS)

Table S2 **Clinical characteristics of study subjects.**
(DOC)

Table S3 **The CSP haplotypes (based on Th2R/Th3R regions) in** ***P. falciparum*** **isolates from Asia, South America and Africa.**
(XLS)
