# Supplementary material for: Genetic Variation in the Plasmodium falciparum Circumsporozoite Protein in India and Its Relevance to RTS,S Malaria Vaccine
Source: PLoS One. 2012 Aug 17;7(8):e43430. doi: 10.1371/journal.pone.0043430 (PMC3422267; doi:10.1371/journal.pone.0043430)
Supplement: Figure S1 — Comparison of Th2R and Th3R sequence polymorphisms in the 1339 global isolates including 179 isolates from current study [Asia, n = 974; South America, n = 181 and Africa, n = 184)]. (DOC) [file pone.0043430.s001.doc]

**Asia South America Africa Total**

Th2R

Th3R

**Fig S1**

**3D7** : NNNNEEPSDKHIKEYLNKIQNSLSTEWSPCSVTCGNGIQVRIKPGSANKPKDELDYANDIEKKI - - - -

**K1** : ............EQ..K...Y..........................D....Q...E....... - - - -

**H1/Dd2** : ............EQ..K.......................................E....... 571 - - 571

H2 : ............EQ..K......................A................E....... 21 - - 21

H3 : ............EQ..K.....................................N.E....... 16 - - 16

H4 : ............EQ..KT..................................Q...E....... 2 - 9 11

H5 : ............EQ.....K..I...............................N.E....... 2 - 7 9

H6 : ............EQ..KN......................................E....... 22 - - 22

H7 : ............EK..KE..................................Q...E....... 9 - - 9

H8 : ................T.....I........................G......N.E....... 6 - - 6

H9 : ............EQ..KE......................................E....... 5 - - 5

H10 : ............EQ..K.......................................K....... 5 - - 5

H11 : ............EQ..KT.K..................................N.E....... 2 - 2 4

H12 : ............EQ..K.....................................V.E....... 4 - - 4

H13 : ............EQ..K.R.....................................E....... 3 - - 3

H14 : .........Q..EK..KT..................................Q...E....... 1 - 1 2

H15 : ............EQ..K.............................S.........K....... 2 - - 2

H16 : ............EQ..KN.....................A................E....... 2 - - 2

H17 : ............EQ..K......................A......V.........E....... 2 - - 2

H18 : ............EQ..K.......................................V....... 1 - - 1

H19 : ........V.PLEQ.FKE......................................E....... 1 - - 1

H20 : ............EQ..K..K...................A................E....... 1 - - 1

H21 : ............EK..KE.....................A................E....... 1 - - 1

H22 : ............EK..KT.....................A................E....... 1 - - 1

H23 : ............EQ..K......................A......G.........E....... 1 - - 1

H24 : ............EQ..K......................A........................ 1 - - 1

H25 : ............E...K.....................................N.E....... 22 - - 22

H26 : ............EK..K.......................................E....... 1 - - 1

H27 : ............EQ..KT.K............................................ 1 - - 1

H28 : ............EQ..K...Y..........................D....Q...E....... 119 - - 119

H29 : ............EQ..K.M.....................................E....... 18 - - 18

H30 : ............T...K..............................G.S......E....... 24 - - 24

H31 : ............T...K.......................................E....... 7 - - 7

H32 : ............EQ..K...Y...................................E....... 3 - - 3

H33 : ............EQ..KN..................................Q...E....... 3 - - 3

H34 : ............EQ..KE..................................Q...E....... 3 - - 3

H35 : ............EQ..K..K..I...............................N.E....... 2 - - 2

H36 : ............EQ..K...................................Q...E....... 2 - - 2

H37 : ............EQ..K..K....................................E....... 1 - - 1

H38 : .............Q..K.......................................E....... 1 - - 1

H39 : ............EQ..K.......................................ED...... 1 - - 1

H40 : ................T.....I........................G......E.E....... 1 - - 1

H41 : ............EQ..K...Y...............................Q...E....... 1 - - 1

H42 : .............Q..T.....I........................G......N.E....... 1 - - 1

H43 : ............TQ..K..............................D........E....... 1 - - 1

H44 : ............TQ..KN.............................G........E....... 1 - - 1

H45 : ............EQ..KN.............................D....Q...E....... 1 - - 1

H46 : ............EQ..KNM.....................................E....... 1 - - 1

H47 : ............EQ..TN.............................D........E....... 1 - - 1

H48 : ............EQ..R.M.....................................E....... 1 - - 1

H49 : ............EQ..RN......................................E....... 1 - - 1

H50 : ............EQ..RN.............................D....Q...E....... 1 - - 1

**H51/MAD20** : ............EQ..K..............................D....Q...E....... 51 - - 51

**H52/7G8** : ............EQ..K..K..I.................................E....... 21 120 4 145

**H53/HB3** : ............EQ..K..............................G.S......E....... 1 42 - 43

H54 : .........Q..EK..KR......................................E....... - 8 - 8

H55 : ............EQ..K....A..............................Q...E....... - 4 - 4

H56 : ............T...KR.............................G.S.NQ...E....... - 1 - 1

H57 : ................KI.............................G................ - 1 - 1

H58 : ............EQ..K....A..............................Q...E....... - 1 - 1

H59 : ............EK..KR......................................E....... - 1 - 1

**H60/3D7**  : ................................................................ - - 24 24

H61 : ............EQ..KR.............................................. - - 17 17

H62 : .........Q..EK..KI..................................Q........... - - 14 14

H63 : .........Q..EK.....K................................Q........... - - 7 7

H64 : .........Q..EK..KT..................................Q........... - - 7 7

H65 : .........Q..EK..KI.K................................Q........... - - 7 7

H66 : .........Q..EK..KT.K................................Q........... - - 6 6

H67 : ............T...KR.............................G.S.N....E....... - 2 3 5

H68 : ............EK..KE.............................G...N....E....... - - 5 5

H69 : .........Q..EK..KR..................................Q........... - - 4 4

H70 : ............T...K..............................G.S.N....E....... - - 3 3

H71 : .........Q..EK..Q...................................Q........... - - 3 3

H72 : .........Q..EK.....K........................D.......Q........... - - 3 3

H73 : ........................................................E....... - - 3 3

H74 : ............EQ.....K..I.............................Q...E....... - - 2 2

H75 : ............EQ..KI.K............................................ - - 2 2

H76 : ............EQ..KR..................................Q........... - - 2 2

H77 : ....................................................Q........... - - 2 2

H78 : ................KI.............................G...E..N.E....... - - 2 2

H79 : .........Q..EK..Q...................................Q.N.E....... - - 2 2

H80 : ............EQ......................................Q........... - 1 1 2

H81 : ......................I.............................Q........... - - 2 2

H82 : .........Q..EK..K...................................Q........... - - 2 2

H83 : ............E...K..K..I.................................E....... - - 2 2

H84 : ............EQ..KT..................................Q........... - - 2 2

H85 : ............EQ..KI.R...........................D....Q........... - - 2 2

H86 : .............Q..K.....I........................D....Q...D....... - - 1 1

H87 : ................KI.............................G................ - - 1 1

H88 : ................KI.P...........................G................ - - 1 1

H89 : ............EQ.....K..I.............................Q........... - - 1 1

H90 : ......................I........................D....Q...I....... - - 1 1

H91 : .........Q..EK..Q..K............................................ - - 1 1

H92 : .............K..KE.............................D...N....E....... - - 1 1

H93 : .........Q..EK..Q..R................................Q.N.E....... - - 1 1

H94 : ............EQ..KT......................................E....... - - 1 1

H95 : .........Q..EQ..KI..................................Q........... - - 1 1

H96 : ............EQ...T......................................E....... - - 1 1

H97 : .............Q..K............................................... - - 1 1

H98 : .............Q..K............................................... - - 1 1

H99 : ........................................G....................... - - 1 1

H100 : ............E...KR.............................................. - - 1 1

H101 : .........Q..EK..KT.K...........................G.S......E....... - - 1 1

H102 : .........Q..EK..Q..K................................Q.N.E....... - - 1 1

H103 : ............EQ..KT.K...............................E....E....... - - 1 1

H104 : .........Q..EK..K..K..I.................................E....... - - 1 1

H105 : .............Q.................................................. - - 1 1

H106 : ................................................................ - - 1 1

H107 : .........Q..EK.....K..I.............................Q........... - - 1 1

H108 : .........Q..EK..KR.............................................. - - 1 1

H109 : .........Q..EK..QI..................................Q........... - - 1 1

H110 : ...................R............................................ - - 1 1

H111 : ..........R..................................................... - - 1 1

H112 : .............Q..KR.............................................. - - 1 1

H113 : .........Q..E...KR..................................Q........... - - 1 1

H114 : ............NK..KR.............................................. - - 1 1

H115 : .........Q..E.........I......................................... - - 1 1

H116 : .........Q..E...K.....I......................................... - - 1 1

H117 : ............EQ..KT.............................................. - - 1 1

**Figure S1:** Comparison of Th2R and Th3R sequence polymorphisms in the 1339 global isolates including 179 isolates from current study [Asia, n=974; South America, n=181 and Africa, n=184)]. The eight laboratory strains are also included in this alignment. These isolates are same as in **Table 2**. A total of 117 haplotypes (H1 to H117) were observed for these 1339 isolates. The haplotype pairs H55 & H58; H57 & H87; H60 & H106 and H97 & H98 are identical at amino acid level; but they differ by one synonymous mutation. H1 to H24 are the same haplotypes we observed in our study sites and shown in **Fig 3B**. MAD20 and Wellcome were identical. Similarly, 3D7 and RO33 were identical. Numbers on the right indicate number of isolates belonging to that particular haplotype reported from Asia, South America and Africa. Dots represent amino acid positions identical to the 3D7 haplotype, whereas those different are indicated. The conserved regions are indicated by gray shading whereas Th2R and Th3R regions have been left un-shaded.
