# Supplementary material for: Genetic Variation in the Plasmodium falciparum Circumsporozoite Protein in India and Its Relevance to RTS,S Malaria Vaccine
Source: PLoS One. 2012 Aug 17;7(8):e43430. doi: 10.1371/journal.pone.0043430 (PMC3422267; doi:10.1371/journal.pone.0043430)
Supplement: Figure S2 — Sliding window (window size = 10 bp, step length = 5 bp) analysis of genetic indices π, Fu & Li's F* and Tajima's D across the Th2R/Th3R region of CSP in populations from (A) Asia, (B) South America and (C) Africa. (DOC) [file pone.0043430.s002.doc]

**Figure S2**

**Fig S2:** Sliding window (window size=10 bp, step length=5 bp) analysis of genetic indices π, Fu & Li’s F* and Tajima’s D across the Th2R/Th3R region of CSP in populations from (**A**) Asia, (**B**) South America and (**C**) Africa. The x-axis represents CSP amino acid positions 305 to 368. The Th2R and Th3R regions are indicated.
