# Supplementary material for: Genetic Variation in the Plasmodium falciparum Circumsporozoite Protein in India and Its Relevance to RTS,S Malaria Vaccine
Source: PLoS One. 2012 Aug 17;7(8):e43430. doi: 10.1371/journal.pone.0043430 (PMC3422267; doi:10.1371/journal.pone.0043430)
Supplement: Text S1 — Information on participants' enrolment, characteristics, and sample collection. (DOC) [file pone.0043430.s003.doc]

**Text S1**

**Supplementary Information on participants’ enrolment, characteristics, and sample collection**

**Cohort study**

This study was carried out from 2005-2010 in the villages of Bargi and Sihora Community Health Centre (CHC) of district Jabalpur. Baseline census of the study villages was conducted to collect demographic and socio-economic information of all the individuals in the study area. A unique identity number is allocated to each individual of the household. Monthly surveillance of fever cases and cases with history of fever was carried out. Current and recent signs and symptoms associated with malaria and anti-malarial treatment history was recorded. Pregnant women were identified and enrolled in the study with or without fever after obtaining written informed consent. Thick and thin film prepared for the diagnosis and differentiation of stages of the malaria parasite. Asexual parasites were counted against 200 WBCs and parasite density per micro litre was calculated by multiplying the count with 40 (multiplication factor), assuming an average individual’s total WBCs count of 8000/µl. After the delivery infants, fathers and siblings were also enrolled and followed up every month for clinical signs and symptoms associated with malaria (longitudinally for three years). When febrile, presence of malaria parasite was checked by light microscopy. Blood samples were collected at the time of enrolment and at the time of malaria infection for further study.

**Hospital study**

Hospital based cross sectional study was conducted at Netaji Subhash Chandra Bose (NSCB) Medical College Hospital at Jabalpur, and Civil Hospital at Maihar (district Satna). Patients with malaria infection diagnosed by microscopy were enrolled in the study after obtaining written informed consent. A structural questionnaire was filled from hospital record of the patient. Blood samples were obtained and processed as described above. Patients were further divided into following categories based on WHO, 2000 classification of malaria severity criteria.

*Uncomplicated malaria (UM)*

Patients who had fever with asexual P. falciparum parasitaemia only in peripheral blood smear examination and had no evidence of severe signs of malaria, no past history of mental/metabolic illness, tuberculosis, meningitis, or accidental head injury were included.

*Cerebral malaria (CM)*

To be considered a case of CM, patient had to fulfill the WHO's definition of CM, have a Glasgow coma score of ≤ 10, peripheral asexual P. falciparum parasitaemia, and have no other clinically evident cause of impaired consciousness, no past history of mental/metabolic illness, tuberculosis, meningitis, or accidental head injury were included.

*Severe Malaria (SM)*

Patients, who satisfy WHO criteria for severe malaria other than cerebral malaria, had no past history of mental/metabolic illness, tuberculosis, meningitis, or accidental head injury.
