# Supplementary material for: Genetic Variation in the Plasmodium falciparum Circumsporozoite Protein in India and Its Relevance to RTS,S Malaria Vaccine
Source: PLoS One. 2012 Aug 17;7(8):e43430. doi: 10.1371/journal.pone.0043430 (PMC3422267; doi:10.1371/journal.pone.0043430)
Supplement: Table S2 — Clinical characteristics of study subjects. (DOC) [file pone.0043430.s005.doc]

**Table S2.** Clinical characteristics of study subjects

| **Variables** | **Cohort Cases** | **Hospital Cases** | | |
| --- | --- | --- | --- | --- |
|  |  | **Uncomplicated Malaria** | **Severe Malaria** | **Cerebral Malaria** |
| Number | 177 | 310 | 69 | 70 |
| Age (IQR) | 24 (8-27) | 23 (13-32) | 22 (12-30) | 19 (10-34) |
| Children, n (%) | 53 (29.9) | 84 (27.1) | 20 (28.9) | 25 (35.7) |
| Gender (M/F) | 44/127 | 160/150 | 41/28 | 35/35 |
| Haemoglobin (g/dl) (IQR) | 9.6 (7.9 – 10.9) | 10.9 (9.3 – 12.6) | 6.8 (5 – 9.9) | 7 (5.2 – 9.2) |
| Seizures, n (%) | 0 | 0 | 9 (13.0) | 13 (18.6) |
| Renal Failure, n (%) | 0 | 0 | 9 (13.0) | 13 (18.6) |
| Jaundice, n (%) | 0 | 0 | 13 (18.8) | 7 (10.0) |
| Respiratory failure, n (%) | 0 | 0 | 2 (2.8) | 7 (10.0) |
| Haemolysis, n (%) | 0 | 0 | 7 (10.1) | 5 (7.1) |
| Shock, n (%) | 0 | 0 | 17 (24.6) | 11 (15.7) |
| Severe Anemia, n (%) | 0 | 0 | 15 (21.7) | 9 (12.9) |
| Multiorgan dysfunction | 0 | 0 | 17 (24.6) | 16 (22.8) |
| Parasite Density per µl | 2420 (720 – 5400) | 960 (400 – 3440) | 1040 (360 – 8000) | 2360 (560 – 19386.6) |

Continuous variables were presented as median (inter quartile range) (IQR) and categorical variables were presented as numbers (%), except gender; Multiorgan dysfunction is defined by combinations of 2 or more complications; Severe and Cerebral Malaria groups had significantly lower haemoglobin (g/dl) than uncomplicated malaria cases (p<0.0001)(t test); Parasite density was significantly higher in Cerebral Malaria group than uncomplicated cases (<0.0001)(t test).
